# Long-term incidence of colorectal cancer after bariatric surgery or usual care in the Swedish Obese Subjects study

**DOI:** 10.1371/journal.pone.0248550

**Published:** 2021-03-25

**Authors:** Magdalena Taube, Markku Peltonen, Kajsa Sjöholm, Richard Palmqvist, Johanna C. Andersson-Assarsson, Peter Jacobson, Per-Arne Svensson, Lena M. S. Carlsson

**Affiliations:** 1 Department of Molecular and Clinical medicine, Institute of Medicine, The Sahlgrenska Academy at University of Gothenburg, Gothenburg, Sweden; 2 Public Health Promotion Unit, National Institute for Health and Welfare, Helsinki, Finland; 3 Department of Neurobiology, Care Sciences and Society, Karolinska Institutet, Solna, Sweden; 4 Department of Medical Biosciences, Pathology, Umeå University, Umeå, Sweden; 5 Institute of Health and Care Sciences, The Sahlgrenska Academy at University of Gothenburg, Gothenburg, Sweden; Weill Cornell Medical College in Qatar, QATAR

## Abstract

Bariatric surgery in patients with obesity is generally considered to reduce cancer risk in patients with obesity. However, for colorectal cancer some studies report an increased risk with bariatric surgery, whereas others report a decreased risk. These conflicting results demonstrate the need of more long-term studies analyzing the effect of bariatric surgery on colorectal cancer risk. Therefore, data from the Swedish Obese Subjects (SOS) study, ClinicalTrials.gov identifier: NCT01479452, was used to examine the impact of bariatric surgery on long-term incidence of colorectal cancer. The SOS study includes 2007 patients who underwent bariatric surgery and 2040 contemporaneously matched controls who received conventional obesity treatment. Patients in the surgery group underwent gastric bypass (n = 266), banding (n = 376) or vertical banded gastroplasty (n = 1365). Information on colorectal cancer events was obtained from the Swedish National Cancer Registry. Median follow-up was 22.2 years (inter-quartile range 18.3–25.2). During follow up there were 58 colorectal cancer events in the surgery group and 67 colorectal cancer events in the matched control group with a hazard ratio (HR) of 0.79 (95% CI:0.55–1.12; p = 0.183). After adjusting for age, body mass index, alcohol intake, smoking status, and diabetes, the adjusted HR was 0.89 (95% CI:0.62–1.29; p = 0.551). When analyzing rectal cancer events separately- 19 events in the surgery group and 31 events in the control group-a decreased risk of rectal cancer with surgery was observed (HR = 0.56; 95% CI:0.32–0.99; p = 0.045, adjusted HR = 0.61 (95% CI:0.34–1.10; p = 0.099), while the risk of colon cancer was unchanged. To conclude- in this long-term, prospective study, bariatric surgery was not associated with altered colorectal cancer risk.

## Introduction

It is well established that the risk of several cancers, including colorectal cancer (CRC), is increased in patients with obesity [1–3]. Bariatric surgery leads to sustained weight-loss, and is associated with reduced overall cancer risk, especially in women [4–6]. Although bariatric surgery has been shown to reduce the risk of obesity-related cancer in general [7], whether the incidence of CRC increases or decreases after this treatment is debated [8].

Retrospective studies in multi-country cohorts have reported an increased risk of CRC with bariatric surgery [9,10]. In contrast, other studies including a meta-analysis of registry-based, retrospective studies have found that bariatric surgery is associated with a decreased risk of CRC [7,11,12]. Factors that may explain these conflicting results include differences in age of patients at inclusion, that various bariatric surgery techniques are used, and choice of statistical method. Given the latency period in colorectal carcinogenesis, the effects of bariatric surgery on subsequent risk of CRC may take decades to become evident and sufficient follow-up periods are therefore also important.

To better understand the effect of bariatric surgery on CRC and other obesity-related types of cancer it is therefore important to use different study populations undergoing various bariatric surgery techniques with long follow-up. To this end, we have examined the long-term incidence of CRC after bariatric surgery and usual care in the Swedish Obese Subjects (SOS) study, an ongoing, matched, prospective, controlled intervention study investigating effects of bariatric surgery.

## Materials and methods

The SOS study protocol, clinical identifier NCT01479452, was approved by seven regional ethics committees in Sweden: Umeå universitet, Medicinska fakulteten, Forskningsetiska kommittén, Karolinska Institutets regionala forskningsetiska kommitté, Lunds Universitet, Medicinska fakultetens forskningsetiska kommitté, Hälsouniversitetet Linköping, Forskningsetiska kommittén, Forskningsetiska kommittén vid Uppsala universitet, Örebro Forskningsetiska kommittén i Örebro, Göteborg Medicinska fakultetens Forskningsetiska kommitté, and written or oral informed consent was obtained from all participants. The ongoing, prospective matched SOS study compares bariatric surgery with conventional obesity treatment [13–16]. In brief, the study enrolled 4047 patients with obesity recruited through campaigns in mass media and at surgical departments and primary health care centres in Sweden between September 1, 1987 and January 31, 2001. Inclusion criteria were age between 37 and 60 years and body-mass index (BMI) (weight in kilograms divided by square of height in meters) of ≥34 for men and ≥38 for women. The exclusion criteria were identical in the treatment groups and aimed to obtain an operable surgical group. The surgery group included 2007 patients of which 376 underwent gastric banding, 1365 vertical banded gastroplasty, and 266 gastric bypass surgery. The control group (n = 2040), received usual obesity treatment and was matched to the surgery group using 18 variables [17]. The intervention study began on the day of surgery for both the surgically treated patient and the matched control patient. Surgery and control patients underwent a baseline examination approximately four weeks before the start of intervention. Clinical examinations were carried out after 0.5, 1, 2, 3, 4, 6, 8, 10, 15 and 20 years. Centralized biochemical examinations were carried out at matching and baseline examinations, and after 2, 10, 15 and 20 years. In addition, questionnaires were filled out at every clinical examination. Colon and rectal cancer events were identified in the Swedish National Cancer Registry using ICD7 codes 153 and 154. The Swedish National Cancer Registry has over 95% coverage for all malignant tumours of which 99% are morphologically verified [18]. In addition, data on death and emigration were obtained by crosschecking social security numbers from the SOS database with the Cause of Death Registry and the Registry of the Total Population. Patients with pre-operative CRC, two controls and one surgery patient, were excluded from the analyses. Kaplan-Meier estimates of cumulative incidence rates were performed to compare time to first cancer diagnosis between the treatment groups. The analyses were performed per-protocol, thus, all participants were included in their original study group until any bariatric surgery was performed in the control group or there was a change in, or removal of, the bariatric surgical procedure in the surgery group, after which they were censored from the analysis. Multivariable Cox proportional hazard models were performed to analyze the impact of potential baseline confounders; age, BMI, alcohol intake, smoking status, and diabetes. The cut-off date for the analysis was December 31, 2018. Median follow-up was 22.2 years (inter-quartile range 18.3–25.2) and follow-up rate was 99.9%.

## Results

At baseline, patients in the surgery group were on average one year younger, but most metabolic risk factors were worse compared to the control group (Table 1).

**Table 1:** Baseline characteristics of the Swedish Obese Subjects study participants*.

Characteristic	Control Group (n = 2038)	Surgery Group(n = 2006)	p-value
Female No (%)	1445 (70.9)	1419 (70.7)	0.908
Age (yr)	48.7±6.3	47.2±5.9	<0.001
Weight (kg)	114.7±16.5	120.9±16.6	<0.001
Height (cm)	169.0±9.2	168.9±9.1	0.587
Body-mass index (kg/m2)	40.1±4.7	42.4±4.5	<0.001
Sagittal diameter (cm)	27.4±3.7	28.9±3.7	<0.001
Blood glucose (mg/dL)	88±32	94±36	<0.001
Serum insulin (mU/l)	18.0±11.4	21.5±13.7	<0.001
Blood pressure (mm Hg)			
Systolic	137.9±18.0	145.0±18.8	<0.001
Diastolic	85.2±10.7	89.9±11.1	<0.001
Lipid levels (mg/dL)			
Total cholesterol	216±42	228±42	<0.001
HDL cholesterol	50±12	54±12	0.845
Triglycerides	177±124	195±133	<0.001
Alcohol consumption (g/day)	5.3±8.1	5.2±7.2	0.632
Daily smoking No (%)	421 (20.8)	518 (25.8)	<0.001
Diabetes at baseline No (%)	262 (12.9)	344 (17.2)	<0.001

Abbreviations: BMI, body mass index (calculated as weight in kilograms divided by height in meters squared); HDL-C, high-density lipoprotein cholesterol.

SI conversion factors: To convert glucose to millimoles per liter, multiply by 0.0555; to convert insulin to picomoles per liter, multiply by 6.945; to convert total cholesterol and HDL-C to millimoles per liter, multiply by 0.0259; and to convert triglycerides to millimoles per liter, multiply by 0.0113.

*Data are presented as mean (SD) unless otherwise indicated.

**Fig 1:**
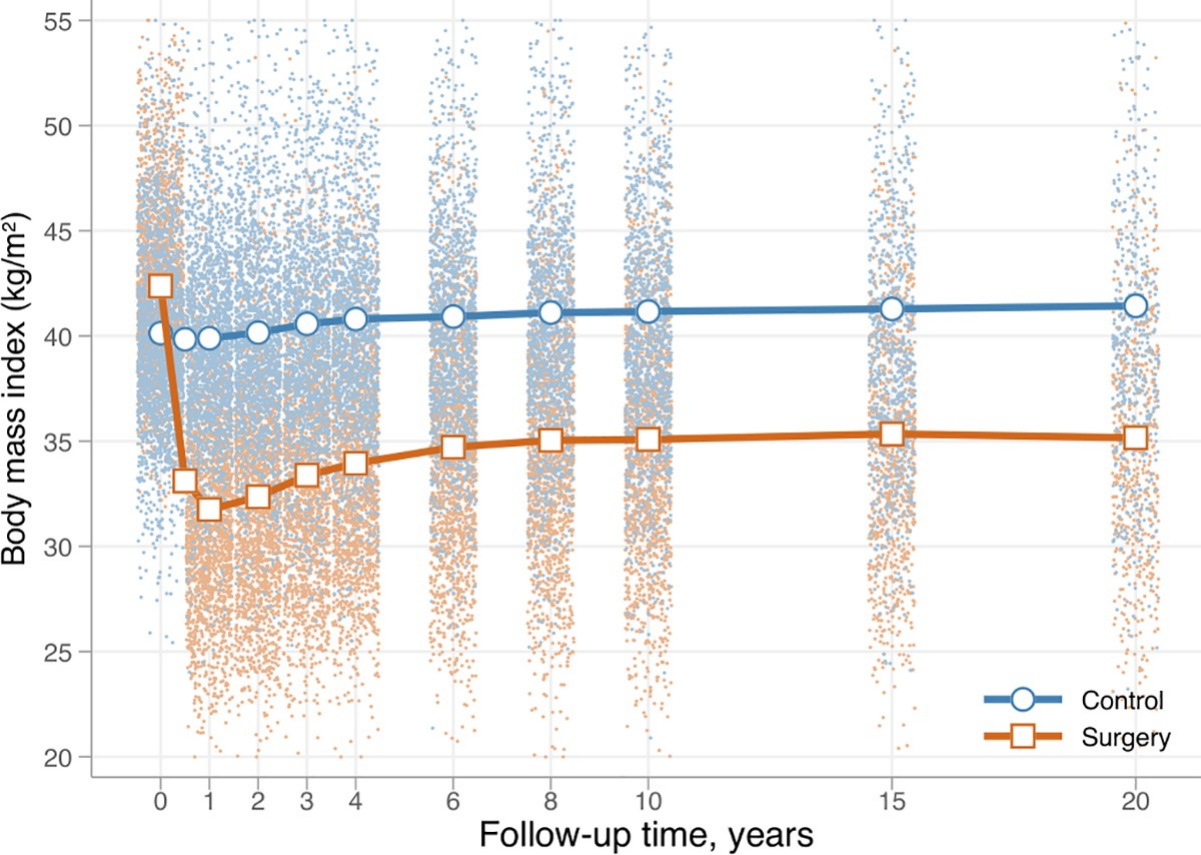
Body mass index in the control and surgery group in the SOS study. Dots represent observed values from individual participants.

After bariatric surgery, mean weight losses (SD) were 28.7 (14.3) kg, 21.1 (15.1) kg, 21.6 (16.6) kg and 22.1 (16.5) kg at the 2-, 10-, 15 and 20-year follow-up visits, respectively (Fig 1).

**Fig 2:**
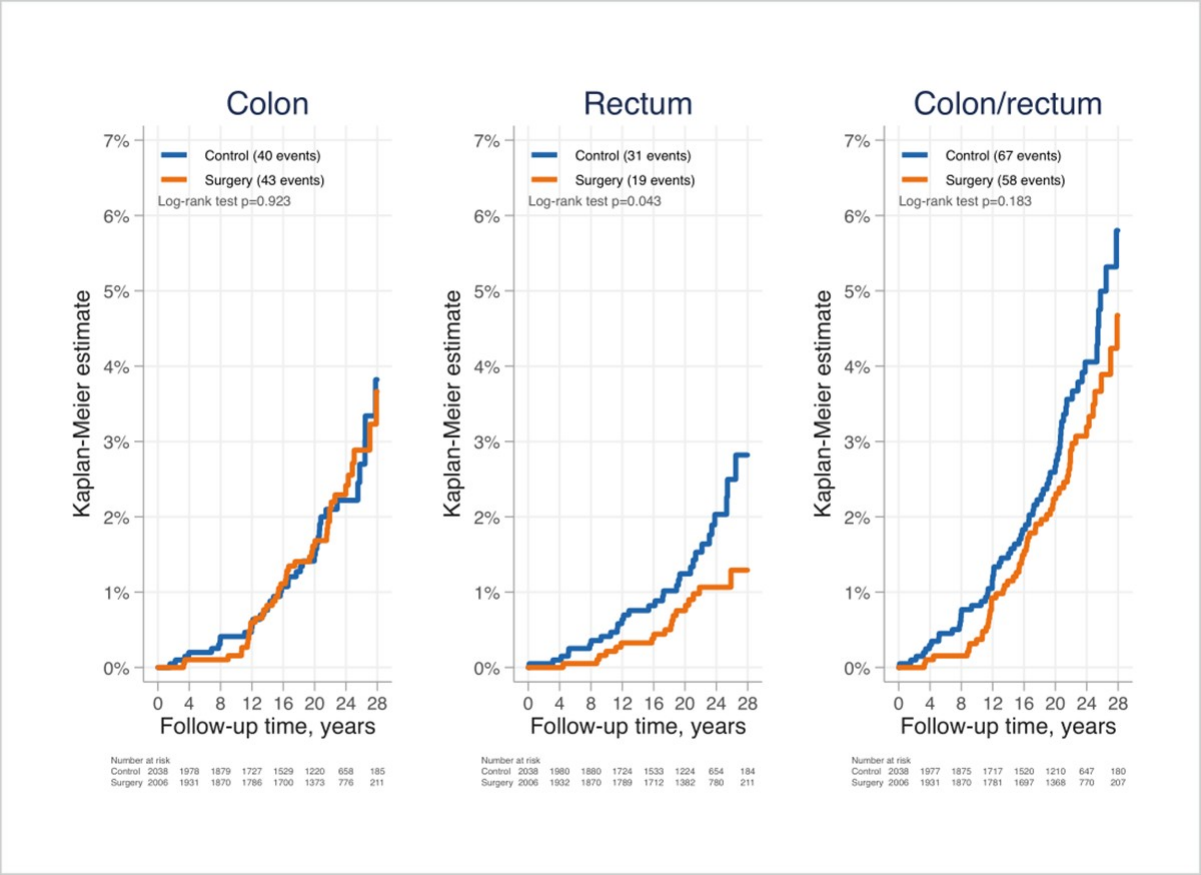
Cumulative incidences of colon cancer (A), rectal cancer (B) and CRC (C) after bariatric surgery or usual care in patients of the Swedish Obese Subjects Study.

The mean weight changes in the control group were small, and never exceeded 3 kg in gain or loss. No differences in incidences of CRC, HR = 0.79 (95% CI:0.55–1.12; p = 0.183), adjHR = 0.89 (95% CI:0.62–1.29; p = 0.551), or colon cancer, HR = 0.98 (95% CI: 0.64–1.50; p = 0.923), adjHR = 1.14 (95% CI:0.72–1.80; p = 0.572) were observed between treatment groups (Fig 2).

However, a decreased risk of rectal cancer in the surgical group was observed, HR = 0.56 (95% CI:0.32–0.99; p = 0.045), but when adjusted for age, BMI, alcohol intake, smoking status and diabetes the statistical significance was lost adjHR = 0.61 (95% CI 0.34–1.10; p = 0.099). In the surgery and control groups, respectively, there were 40 and 45 CRC events in women (adjHR = 0.89, 95% CI:0.57–1.40; p = 0.615) and 18 and 22 CRC events in men (adjHR = 0.90, 95% CI:0.48–1.69; p = 0.747).

## Discussion

Data from the prospective SOS-study, which compares a bariatric surgery group with a matched control group, could not confirm that bariatric surgery is associated with an altered risk of CRC. However, in an unadjusted analysis, we observed a decreased risk of rectal cancer with surgery. This is in line with Christou *et al*, reporting a clinically important, relative risk of CRC with surgery of 0.32 (95% CI .076–1.313; p = 0.063) [19]. Future studies with larger cohorts and a greater number of rectal cancer events may strengthen this finding.

We can only speculate about the differences between the SOS study and recent retrospective studies reporting either increased or decreased risk of CRC following bariatric surgery [8]. New surgery techniques have been developed during the long follow up period. It is possible that various bariatric surgery techniques may affect the colonic epithelium, and thereby the expression of mucosal CRC biomarkers, in different ways. It has been reported that pro-inflammatory and tumorigenic COX-1 levels increase 6 months after Roux-en-Y gastric bypass (RYGB) surgery with a biliopancreatic limb of 150 cm [20], but decreases after RYGB with a shorter biliopancreatic limb [21]. In addition, COX-1 levels are unchanged after sleeve gastrectomy [22]. However, as many environmental and lifestyle factors influence the CRC risk, a controlled study design is probably of utmost importance. In the studies reporting an increased risk of CRC with bariatric surgery, known confounders such as smoking or BMI were unknown in the control groups. This may be problematic, not only as BMI is a significant variable that may affect the assessment of surgery outcomes, but also because of the known association between BMI and colon cancer [3]. Future studies exploring altered CRC risk with bariatric surgery should ensure adequate statistical power, either with larger study sizes or higher incidence rates, and diverse bariatric surgery techniques. Nonetheless, it should be noted that in general, bariatric surgery appears to reduce cancer risk and increase longevity [4,5,23].

The SOS study has some limitations. A majority of the patients in the surgery group underwent vertical banded gastroplasty or banding, methods rarely used today. In addition, cancer incidence was not a predefined endpoint, and the study was therefore not designed to address the current research question. Hence, despite a large number of patients and very long follow-up, the number of CRC events was limited. Strengths include the prospective design, the well-characterized matched control group, and the possibility to crosscheck social security numbers of participants with almost 100% complete national registers.

In conclusion, in this prospective controlled study, we could not verify that bariatric surgery is associated with changes in risk of CRC in patients with obesity.
